# On the Use of Blood Samples for Measuring DNA Methylation in Ecological Epigenetic Studies

**DOI:** 10.1093/icb/icaa123

**Published:** 2020-08-24

**Authors:** Arild Husby

**Affiliations:** Evolutionary Biology, Department of Ecology and Genetics, Uppsala University, SE-75236 Uppsala, Sweden

## Abstract

There is increasing interest in understanding the potential for epigenetic factors to contribute to phenotypic diversity in evolutionary biology. One well studied epigenetic mechanism is DNA methylation, the addition of a methyl group to cytosines, which have the potential to alter gene expression depending on the genomic region in which it takes place. Obtaining information about DNA methylation at genome-wide scale has become straightforward with the use of bisulfite treatment in combination with reduced representation or whole-genome sequencing. While it is well recognized that methylation is tissue specific, a frequent limitation for many studies is that sampling-specific tissues may require sacrificing individuals, something which is generally undesirable and sometimes impossible. Instead, information about DNA methylation patterns in the blood is frequently used as a proxy tissue. This can obviously be problematic if methylation patterns in the blood do not reflect that in the relevant tissue. Understanding how, or if, DNA methylation in blood reflect DNA methylation patterns in other tissues is therefore of utmost importance if we are to make inferences about how observed differences in methylation or temporal changes in methylation can contribute to phenotypic variation. The aim of this review is to examine what we know about the potential for using blood samples in ecological epigenetic studies. I briefly outline some methods by which we can measure DNA methylation before I examine studies that have compared DNA methylation patterns across different tissues and, finally, examine how useful blood samples may be for ecological studies of DNA methylation. Ecological epigenetic studies are in their infancy, but it is paramount for the field to move forward to have detailed information about tissue and time dependence relationships in methylation to gain insights into if blood DNA methylation patterns can be a reliable bioindicator for changes in methylation that generate phenotypic variation in ecologically important traits.

## Introduction

Over the past few years, there has been a tremendous increase in the number of studies that examine epigenetic mechanisms in natural populations (e.g., Shindo et al. 2006; Wenzel and Piertney 2014; Colicchio et al. 2015; Platt et al. 2015; Baerwald et al. 2016; Laine et al. 2016; Saino et al. 2017; Liu et al. 2019a; Viitaniemi et al. 2019; Heckwolf et al. 2020). Common to all these studies is the wide range of tissue types that have been used, and in particular, the widespread use of blood samples (see Table 1). In this review, I focus on what we know about DNA methylation patterns in blood and how they compare to DNA methylation patterns in other tissues. Does changes in DNA methylation measured from the blood have functional (i.e., phenotypic) consequences? Do DNA methylation patterns in blood samples resemble that seen in other, perhaps often more important, tissues? More and more ecological epigenetic studies are reported as sequencing costs are declining and many studies use blood as a tissue type: this review is to draw attention to carefully consider the type of tissue we use in ecological epigenetic studies and some of the challenges, but also opportunities, that come with using blood as a tissue type.

**Table 1:** Examples of evolutionary ecological studies that have examined DNA methylation in vertebrate populations, the phenotype, and the tissue used

References	Organism	Method	Genomic coverage	Phenotype	Tissue type
Hu et al. 2018	Guppy (*Poecilia reticulata*)	RRBS	Genome wide	Infection to *Gyrodactylus*	Skin
Gore et al. 2018	Mexican cavefish (*A. mexicanus*)	WGBS	Genome wide	Eye degeneration	Eye
Heckwolf et al. 2020	Three-spined stickleback (G*asterosteus aculeatus*)	WGBS, RRBS	Genome wide	Salinity tolerance	Gill
Navarro-Martin et al. 2011	European Sea bass (*D. labrax*)	Pyrosequencing	Candidate gene	Temperature dependent sex ratio shifts	Gonads
Riyahi et al. 2015	Great tit (*P. major*)	Pyrosequencing	Candidate gene (SERT and DRD4)	Novelty seeking behaviour	Whole blood
Schrey et al. 2012	House sparrow (*Passer domesticus*)	MS-AFLP	Genome wide	Population expansion	Whole blood
Wenzel and Piertney 2014	Red grouse (*Lagopus lagopus scotica*)	MS-AFLP	Genome wide	Gastrointestinal parasite load	Liver
Viitaniemi et al. 2019	Great tit (*P. major*)	RRBS	Genome wide	Seasonal reproduction	Red blood cells
Rubenstein et al. 2016	Superb starling (*Lamprotornis superbus*)	Pyrosequencing	Candidate gene (NR3C1)	Probability to breed and disperse	Whole blood
Derks et al. 2016	Great tit (*P. major*)	WGBS	Genome wide	NA	Whole brain and whole blood
Verhulst et al. 2016	Great tit (*P. major*)	Pyrosequencing	Candidate gene (DRD4)	personality	Brain (hypothalamus, hippocampus), Whole blood
Merondun et al. 2019	Canadian lynx (*Lynx canadensis*)	EpiGBS	Genome wide	Epigenetic structure	Epidermal tissue
Taff et al. 2019	Tree swallows (*Tachycineta bicolor)*	Methylated DNA immunoprecipitation (MeDIP)	Genome wide	Plumage color and stress response	Whole blood
Saino et al. 2017	Barn swallow (*Hirunda rustico*)	Pyrosequencing	Candidate gene (CLOCK)	Departure from wintering grounds, arrival on breeding grounds, timing of reproduction, breeding success	Whole blood
Watson et al. 2019	Great tit (*P. major*)	Methylation sensitive immunoabsorbent assay	Genome wide	Ontogentic plasticity	Whole blood and whole embryos
Lea et al. 2016	Yellow baboon (*Papio cynocephalus*)	RRBS	Genome wide	Resource availability	Whole blood

Ecological epigenetic studies are of rising interest, no doubt facilitated by decline in sequencing costs and the development of new methods to measure epigenetic mechanisms such as chromatin modification (Assay for Transposase Accesible Chromatin using sequencing, or so called ATAC sequencing), non-coding RNAs and DNA methylation (bisulfite sequencing). While epigenetic modifications such as DNA methylation, histone modification and small RNA are interesting to study in their own right, of particular interest to evolutionary ecologists is the potential for epigenetic variation to contribute to phenotypic diversity in ecologically important traits within and between populations (Merot et al. 2020).

There is little doubt that epigenetic mechanisms can contribute to phenotypic variation at this point: in toadflax (*Linaria vulgaris*) two different flower phenotypes exists where the mutant has radial rather than bilateral flower arrangement and this variation is due to DNA methylation at the *cycloidea* gene (Cubas et al. 1999). In Mexican cavefish (*Astyanax mexicanus*), the blind cave morph is a result of DNA methylation at several known eye development genes and that the observed differences in DNA methylation are causally involved and have been confirmed with functional studies on zebrafish (Gore et al. 2018). In Siberian hamsters (*Phodopus sungorus*), changes in hypothalamic DNA methylation at the *dio3* gene are involved the seasonal response to daylength (Stevenson and Prendergast 2013).

These are just a few examples of the wide diversity of both traits and organisms where phenotypic variation is, at least in part, a result of differences in DNA methylation at individual loci. More controversial remains the stability of epigenetic marks and thus their potential long-term evolutionary consequences (Skipper 2011; Grossniklaus et al. 2013; Donohue 2014).

A notable observation from ecological epigenetic studies is that, as a brief look at Table 1 also makes clear, the type of tissue examined varies widely and many epigenetic studies on vertebrates use blood as tissue type. But what do we actually know about the functional relevance of DNA methylation changes measured in blood? This article focuses specifically on the question of whether observed patterns of DNA methylation measured in the blood can be representative of DNA methylation patterns in other tissues and I review recent studies that have addressed this question.

### What is DNA methylation and why study it?

Epigenetic mechanisms are increasingly recognized as an important factor controlling gene regulation and one of the most examined epigenetic mechanisms is DNA methylation: the addition of a methyl group to the 5′ position of cytosine residues in the genome. This can take place either in the context of CpG sites, CHG, or CHH (H being either A, T, or C). The type of DNA methylation depends both on tissue as well as organism, for example, in plants CHH methylation is common, but in mammals and birds, DNA methylation occurs mainly in a CpG context (Suzuki and Bird 2008). DNA methylation is typically associated with repressed gene expression but this depends on the genomic region and the exact mechanism by which occur is not yet fully understood (Luo et al. 2018), and can, therefore, have important functional consequences.

### How can we measure DNA methylation?

Measuring DNA methylation has historically been done using methylation sensitive AFLP (MS-AFLP) in non-model organisms whereby two restriction enzymes are used (e.g., MSPI and HPAll which are DNA methylation insensitive and sensitive, respectively) to simultaneously screen a large number of loci (e.g., Schrey et al. 2012; Liebl et al. 2013). While this is an efficient and inexpensive method to obtain genome-wide DNA methylation information, it cannot be used to obtain information about which genes, let alone which CpG sites, are methylated or not since the nearby sequence to the site targeted is unknown. Thus, while providing a useful overview of global methylation levels, this method poses obvious challenges when trying to move into the functional consequences of changes in methylation.

The gold standard technology for detection of 5mC DNA methylation is bisulfite sequencing which provides quantitative information about DNA methylation at individual CpG sites (Suzuki and Bird 2008). Following bisulfite conversion of the DNA cytosines is deaminated to give uracil unless methylated. Therefore any cytosines that remain in bisulfite-treated DNA must have been methylated. The most comprehensive assay of DNA methylation patterns is the use of whole genome bisulfite sequencing (WGBS) but also reduced representation approaches (RRBS) provide genome wide information although this method is biased toward information from particular genomic regions such as CpG islands and promoters (Gu et al. 2011).

It is worth noting that the bisulfite treatment of the DNA itself can lead to fragmentation and degradation of the DNA, which may impact downstream analyses. Newer methods that side-step the bisulfite treatment has been developed by using TET-assisted pyridine borane sequencing and show great promise (Liu et al. 2019b). I am not aware that this has been used in the context of ecological epigenetics yet, however. It should also be noted that SMRT (PacBio) sequencing will directly obtain information about methylation due to differences in the kinetic energy of the different base modifications (Flusberg et al. 2010). There are many excellent reviews covering the technical details of how to obtain DNA methylation information from the different sequencing platforms and how to analyze such data and I refer the reader to these for further details (Suzuki and Bird 2008; Zentner and Henikoff 2014; Ziller et al. 2015; Lea et al. 2017; Teschendorff and Relton 2018; Sepers et al. 2019).

### DNA methylation is tissue and cell specific

The most detailed information about differences in DNA methylation across tissues comes from human studies. Notably, the National Institute of Health Roadmap Epigenomics Consortium has published an overview of the genetic and epigenomic patterns across cell and tissues in 111 different human reference genomes (Roadmap Epigenomics Consortium 2015). One of the important insights from this work is that different cell types within a tissue type can display more similar methylation profiles with cell types of shared developmental origin in another tissue than other cells within the tissue. That is, within tissues there can be large heterogeneity in methylation levels depending on the origin of the cells making up that tissue (Roadmap Epigenomics et al. 2015). Differentially methylated sites among tissues are often related to tissue-specific functions and in particular, hypomethylated differentially methylated regions (DMRs) frequently overlap with tissue-specific enhancers (Schultz et al. 2015). This is important because DNA methylation levels in tissue-specific DMRs are negatively correlated with expression levels, particularly around the transcription start site (Schultz et al. 2015) and thus likely have functional significance.

Tissue (and cell)-specific DNA methylation patterns are thus ubiquitous (Wan et al. 2015) and such patterns can even be conserved across species (Zhou et al. 2017). For example, comparing rat and human blood, 11% of tissue-specific methylation DMRs are conserved, whereas this increases to 27% when comparing the more closely related species mouse and rat (Zhou et al. 2017). Similarly, Blake et al. (2020) also find larger differences in DNA methylation between tissues than across human, chimpanzee, and rhesus macaque.

Methylation comparison across tissues has also been done in a study of European sea bass (*Dicentrarchus labrax*) where individuals were experimentally exposed to high temperatures during early development which resulted in an increase in DNA methylation at the cyp19a promoter region (resulting in a greater proportion of males)(Navarro-Martin et al. 2011). This methylation change was only observed in gonadal tissue and not in the brain and, interestingly, was only observed at the promoter of the *cyp19a* gene and not at a housekeeping gene (Navarro-Martin et al. 2011), again demonstrating that DNA methylation levels can be both tissue and gene specific.

A key issue to address is whether there are conserved methylation patterns across tissues (within species) and, in particular, if DNA methylation patterns measured in blood reflect that in other tissues since ecological epigenetic studies are often only able to sample peripheral tissues such as blood in birds and mammals.

### DNA methylation patterns in brain and blood

If blood could be used as a reliable tissue type for DNA methylation patterns in inaccessible but more relevant tissues, such as areas of the brain, this would obviously significantly ease sampling. A few studies have examined this and have found mixed results. Siller and Rubenstein (2019) were interested in methylation in the promoter region of the glucocorticoid receptor gene (part of the vertebrate stress response) in the hippocampus and hypothalamus in European starlings and how this compares to that found in the blood. As expected, DNA methylation levels differed among these tissues but there was no correlation between methylation levels observed in the blood and the different areas of the brain. Thus, at least for the promoter region of this gene blood does not seem to be useful as a biomarker for DNA methylation in the brain areas examined.

Laine et al. (2016) used WGBS to measure methylation in whole brain and whole blood of a single male great tit (*Parus major*). Examining 10.2 million CpG sites in the two tissue types they observe reduced CpG methylation within CpG islands and around the transcription start site (TSS) in both tissues and, consistent with these findings, there was reduced gene expression in the brain (no expression data were available for blood) within gene bodies and around the TSS. In a follow-up study on the same individual, Derks et al. (2016) show that there are distinct differences between CpG and non-CpG (CHH and CHG) methylation in the brain and the blood, average CpG methylation level in the brain was higher and there was essentially no non-CpG methylation in blood but low levels in the brain. Derks et al. also examined sites that were differentially methylated between the two tissues in different genomic features (TSS, gene body, and TTS) and found a few hundred sites, of which many were enriched for biological processes related to the specific function of the two tissue types examined. This result is not surprising since tissue-specific DNA methylation is well known to be involved in tissue specialization (as discussed above).

It would be very useful to have information on sites or regions where DNA methylation is consistent between tissues within individuals but differ between individuals and thus might, therefore, contribute to observe between individual phenotypic differences. To my knowledge, this has not yet been done in ecological studies, but there is one such recent study on humans. Gunasekara et al. (2019) used samples from the NIH genotype-tissue expression study to identify correlated genomic regions where between individual variation in DNA methylation occurs systemically. Such regions are of great interest because of the potential for profiling more peripheral tissues to obtain informative DNA methylation patterns that are involved in regulating expression in a concerted fashion within an individual but that differs between individuals. Gunasekara et al. (2019) found 9926 such genomic regions in the human genome (comprising 0.1% of the human genome) for the thyroid, heart, and brain tissue that they profiled and they were associated with genes implicated in a broad range of phenotypes (and diseases). An important finding was that DNA methylation in one tissue correlated not only with gene expression at that tissue for these regions but, importantly, also at other tissues.

This study highlights the potential for using non-invasive tissue samples, such as blood, to provide insights into the epigenetic regulation in other tissues. Similar studies on ecological model organisms would thus be highly useful. Moreover, if such regions are conserved across species (as seen above for tissue-specific DMRs in the study by Zhou et al. 2017) such regions could potentially be used across species (of course independent validation and replication would be needed first).

### DNA methylation patterns in blood

Many ecological epigenetic studies on vertebrates use blood samples as tissue types when assessing DNA methylation patterns (Table 1). This is not surprising given the ease at which this tissue type can be sampled. However, blood as tissue consists of many different cell types (Scanes 2015) and there are also taxonomic differences in cell composition. For example, in birds, the mature red blood cells are nucleated and thus contain DNA (Scanes 2015), whereas this is not the case in mammals. Moreover, the longevity of avian erythrocytes is approximately half that compared their mammalian counterparts (Scanes 2015). These taxonomic differences and differences in temporal dynamics of cell longevity are important to keep in mind when using blood as tissue in DNA methylation studies. Also within species, there can be differences in the cell composition of blood over the season (Pickering 1986) and a consequence of such cell-specific changes in composition of whole blood, combined with the fact that there are different DNA methylation profiles in different cells (Schultz et al. 2015), is that this can lead to observed changes in DNA methylation which may potentially lead to confounding signals. For example, one might take a blood sample of an individual before and after exposure to some immune challenge and look at DNA methylation differences in relation to this treatment from blood samples. The observed change in DNA methylation at individual CpG sites within genes might then be a result of changes in the ratio of different cell types prior to and after treatment (e.g., white blood cells might increase following the immune challenge) and instead reflect differences in methylation profiles of different cell types, rather than an adaptive change in methylation in response to the immune challenge. This obviously complicates the inferences one can draw about the functional role of DNA methylation changes on the phenotype (but note that changes in cell type composition are interesting to study in their own right). Controlling for cell-type heterogeneity is therefore an important but challenging aspect for ecological epigenetic studies. In a recent review on challenges with analyzing ecological and evolutionary epigenetic studies, Lea et al. (2017) argues there are three main ways to approach this issue: first, one could take advantage of information about cellular composition of the tissue examined (say from microscopy samples) and include such information in the statistical models. Second, if no such information is available one could use epigenomic profiles from sorted cell types to predict the composition of samples that have mixed cell types. Epigenomic profiles from cell types are available from several organisms and, Lea et al. (2017) argues, could potentially be used across species. There certainly does seem to be some methylation marks that are stable across organisms (e.g., Blake et al. 2020) but I would caution against such use unless this has been specifically and extensively verified for the specific organism of interest. Moreover, given the evolutionary conserved status of such methylation marks it seems likely these are not of main interest for generating phenotypic diversity within species. Cell type-specific epigenomic profiles could be used to examine if sites that are differentially methylated with respect to phenotype of interest are differentially methylated by cell type and thus potentially be a result of confounding by cell composition variation. Finally, there are statistical methods being developed that allow one to control for cell type composition in the absence of information on epigenomic cell type data, but at least so far these methods have lower power than reference-based approaches (Lea et al. 2017).

I am aware of only one ecological study on DNA methylation that has attempted to control for cell-type heterogeneity. In a study on the effect of resource availability on DNA methylation in yellow baboons (*Papio cynocephalus*), Lea et al. (2016) used information on cell-type proportion data from blood smears in the same population and cell type-specific DNA methylation data from human whole blood to test if resource availability predicted cell-type composition effects and if sites associated with resource availability are more likely to exhibit cell type-specific DNA methylation patterns. In this specific case, the results were unlikely to be explained by cell type-specific heterogeneity effects.

An alternative to statistically control for cell-type heterogeneity in sampled tissue is to, if possible, use cell sorting methods to obtain DNA methylation information from specific cell types. A few ecological epigenetic studies have done this. For example, Viitaniemi et al. (2019) examined temporal changes in DNA methylation over the breeding season in great tits using red blood cells instead of whole blood (Makinen et al. 2019), and Pértille et al. (2017) examined changes in red blood cell methylation in chicken in relation to rearing conditions. Obviously, if using cell-specific methylation information, another question that arises is which cells are relevant to sample with respect to the phenotype studied.

### Correlations between changes in DNA methylation across tissues

Epigenetic mechanisms are thought to play an important role in environmentally induced changes in phenotypes (i.e., phenotypically plastic traits) (Flores et al. 2013; Kilvitis et al. 2017). In such cases, environmentally induced changes in DNA methylation should alter gene regulation and thus the phenotype in question, something that is expected to happen within the particular tissue that regulates the behavior. This is what has been observed in seasonal timing of reproduction in Siberian hamsters for example where a change in DNA methylation at the *dio2* gene within the hypothalamus is involved in regulation of gonadal growth and recession in response to differences in photoperiod (Stevenson and Prendergast 2013). While we know that there are correlations in DNA methylation patterns across tissue the question now becomes whether we can detect changes in DNA methylation in one tissue, as the hypothalamus in the hamster example, in peripheral tissue such as blood. We have very little information about this. In great tits, there does seem to be some indication that this might be possible. Lindner et al. (in review) sampled great tit blood, liver, gonads, and hypothalamus from different timepoints and individuals to examine the correlation between a change in DNA methylation across timepoints in the liver and the change in DNA methylation across the same timepoints in red blood cells. Out of a total of 302,000 GpG present in both tissues at >10× coverage across time periods, around 2500 and nearly 4000 CpG sites showed differential methylation in either or both of the two tissues for the two time points. When examining the correlation in change in DNA methylation at individual CpG sites between timepoints across the two tissues, there was a strong and positive correlation both at the promoter region and in the TSS (*r *≈ 0.6–0.8). Thus, changes in DNA methylation over time in liver was strongly reflected by changes in DNA methylation in red blood cells, also when restricting this analysis to sites just located within the promoter region. This analysis provides important information that, at least for these two tissues in this species and time span, it is possible to detect changes in DNA methylation in one tissue (liver) from DNA methylation patterns in red blood cells. It is possible that, similar to the cross-tissue correlations in methylation levels observed in different species, there is also potential for tissue-specific effects where temporally or environmentally induced changes in DNA methylation can be detected in blood samples. If this is a general finding this would open the possibility to probe tissue specific changes in methylation from taking repeated blood samples from individuals. Although this would be very exciting, much more work is needed to confirm these findings in other tissues and species and, not least, link changes in methylation to changes in expression. For example, Lindner et al. (in review) did not find a correlation between the observed change in DNA methylation in liver between timepoints and a corresponding change in gene expression within the same tissue, although power was low as there were few CpG sites within the promoter region of each gene. Moreover, Lindner et al. (in review) unfortunately did not have methylation data from gonads and hypothalamus so could not examine if changes in DNA methylation were related to changes in expression in these tissues.

An additional complication for gaining insights into the functional consequences of changes in DNA methylation in general, and particularly for tissue general co-methylation changes, is that we do not yet have a good understanding of when changes in DNA methylation at individual CpG site or DNA methylation at larger regions are important. As the functional significance of DNA methylation is highly dependent on genomic context (Suzuki and Bird 2008), it seems likely that also the functional consequence of DNA methylation change at individual CpG sites or regions will depend on the genomic context.

### Outlook on ecological epigenetics and the use of blood samples as tissue type

The ease at which genome wide DNA methylation information can be obtained should not be substituted for careful experimental design and sampling. An open question is if DNA methylation patterns in blood samples can be informative for methylation changes in more relevant tissues and thus have functional consequences. It is clear that correlations in DNA methylation levels as well as changes in DNA methylation levels across tissues, including blood, exist and examining the mechanism behind these correlations will provide useful information for future epigenetic studies.

One exciting future avenue for ecological epigenetic studies is the potential for using cell free DNA from blood samples. Cell-free DNA (cfDNA) are small fragments of DNA present in the blood, urine, and other body fluids released by the host cells or microbial cells (Burnham et al. 2016). Several recent studies on humans have demonstrated that sequencing of methylation from cfDNA in blood can provide a rapid, noninvasive and, not least, an easily repetitive method for providing information on the status on a range of different phenotypes such as infection responses to injury (Cheng et al. 2019), autoimmune diseases, tumors and even chromosomal abnormalities in fetuses (Fan et al. 2008). The origin of cfDNA can be inferred based on the DNA methylation profile of cells and thus this method can potentially become an important tool for tracking DNA methylation changes in particular tissues for ecological epigenetic studies in the future.

## Notes

From the symposium “Epigenetic Variation in Endocrine Systems” presented at the annual meeting of the Society for Integrative and Comparative Biology, January 3–7, 2020 at Austin, Texas.
